# Dexmedetomidine Use in Infants Undergoing Cooling Due to Neonatal Encephalopathy (DICE Trial): A Randomized Controlled Trial: Background, Aims and Study Protocol

**DOI:** 10.3389/fpain.2021.770511

**Published:** 2021-12-07

**Authors:** Mariana Baserga, Tara L. DuPont, Betsy Ostrander, Stephen Minton, Mark Sheffield, Alfred H. Balch, Timothy M. Bahr, Kevin M. Watt

**Affiliations:** ^1^Division of Neonatology, Department of Pediatrics, University of Utah, Salt Lake City, UT, United States; ^2^Division of Neurology, Department of Pediatrics, University of Utah, Salt Lake City, UT, United States; ^3^Intermountain Healthcare, Provo, UT, United States; ^4^Division of Pediatric Clinical Pharmacology, University of Utah, Salt Lake City, UT, United States; ^5^Division of Pediatric Clinical Pharmacology and Division of Critical Care, University of Utah, Salt Lake City, UT, United States

**Keywords:** hypoxia-ischemia encephalopathy, therapeutic hypothermia, neuroprotection, pain, sedation, morphine, dexmedetomidine

## Abstract

**Background:** Neonatal hypoxia-ischemia encephalopathy (HIE) is the leading cause of neonatal death and poor neurodevelopmental outcomes worldwide. Therapeutic hypothermia (TH), while beneficial, still leaves many HIE treated infants with lifelong disabilities. Furthermore, infants undergoing TH often require treatment for pain and agitation which may lead to further brain injury. For instance, morphine use in animal models has been shown to induce neuronal apoptosis. Dexmedetomidine is a potent α_2_-adrenergic receptor agonist that may be a better alternative to morphine for newborns with HIE treated with TH. Dexmedetomidine provides sedation, analgesia, and prevents shivering but does not suppress ventilation. Importantly, there is increasing evidence that dexmedetomidine has neuroprotective properties. Even though there are limited data on pharmacokinetics (PK), safety and efficacy of dexmedetomidine in infants with HIE, it has been increasingly administered in many centers.

**Objectives:** To review the current approach to treatment of pain, sedation and shivering in infants with HIE undergoing TH, and to describe a new phase II safety and pharmacokinetics randomized controlled trial that proposes the use of dexmedetomidine vs. morphine in this population.

**Methods:** This article presents an overview of the current management of pain and sedation in critically ill infants diagnosed with HIE and undergoing TH for 72 h. The article describes the design and methodology of a randomized, controlled, unmasked multicenter trial of dexmedetomidine vs. morphine administration enrolling 50 (25 per arm) neonates ≥36 weeks of gestation with moderate or severe HIE undergoing TH and that require pain/sedation treatment.

**Results and Conclusions:** Dexmedetomidine may be a better alternative to morphine for the treatment of pain and sedation in newborns with HIE treated with TH. There is increasing evidence that dexmedetomidine has neuroprotective properties in several preclinical studies of injury models including ischemia-reperfusion, inflammation, and traumatic brain injury as well as adult clinical trials of brain trauma. The Dexmedetomidine Use in Infants undergoing Cooling due to Neonatal Encephalopathy (DICE) trial will evaluate whether administration of dexmedetomidine vs. morphine is safe, establish dexmedetomidine optimal dosing by collecting opportunistic PK data, and obtain preliminary neurodevelopmental data to inform a large Phase III efficacy trial with long term neurodevelopment impairment as the primary outcome.

## Introduction

Neonatal hypoxia-ischemia encephalopathy (HIE) is the leading cause of neonatal death and poor neurodevelopmental outcomes worldwide (1–3). Up to 12,000 infants are affected each year in the US (2). Therapeutic Hypothermia (TH) initiated within 6 h of life has become standard of care in developed countries and has been shown to mitigate brain damage (4–6). However, current data from the more recent cooling trials demonstrate that up to 30% of treated infants either died or had moderate to severe neurologic disabilities including long-term motor and cognitive dysfunction (7). Disabilities include cerebral palsy, intellectual disability, epilepsy and visual impairment among others adverse outcomes. No additional therapies have yet proven to be efficacious in further reducing brain injury and impairment for these high risk infants.

Newborns with moderate-to-severe HIE may also present with multiorgan failure and may develop cardiovascular instability, renal and liver insufficiency, seizures and respiratory failure requiring mechanical ventilation. Due to their critical condition in the Neonatal Intensive Care Unit (NICU) they commonly receive opiate drugs such as morphine for pain and sedation management and to prevent shivering (8, 9).

The present review will evaluate the current approach to treatment of pain, sedation and shivering in infants with HIE undergoing TH, and will describe a new phase II safety and pharmacokinetics (PK) randomized controlled trial that proposes the use of dexmedetomidine vs. morphine in this population.

### Adequate Pain and Sedation Management May Improve Outcomes

In neonates with HIE who are treated with TH, emerging data suggest that appropriate treatment of pain and agitation may improve outcomes (10–12). Infants with HIE are subjected to the stresses of an adverse perinatal event, resuscitation, hypothermia itself and painful procedures. These events may alter nociceptive pathways during a critical developmental period and adversely affect neuropsychological outcomes (13). Thoresen et al reported that TH was *not* protective after HIE in unsedated piglets, but TH reduced the severity of brain injury in piglets receiving appropriate sedation (14). However, in most trials of TH for neonatal HIE the administration of sedation drugs was provider-driven and there have not been any large randomized controlled trial addressing this important question (8, 9).

### Morphine Is the Most Common Sedative Used in Neonates With HIE During Therapeutic Hypothermia

A recent study including 125 neonatal intensive care units (NICUs) demonstrated that up to 64% of neonates with HIE and treated with TH received opioids (15). Unpublished data from the ongoing High Dose Erythropoetin for Asphyxia and Encephalopathy (HEAL) trial showed that 328/501 (65%) infants with HIE received morphine in the first 4 days of life at 17 participating NICUs (NCT02811263).

However, the use of morphine for pain and sedation has raised concerns for safety including depressed ventilation, hypotension, gastrointestinal dysmotility and importantly, potential adverse effects on neurodevelopmental outcomes (10–12). For instance, morphine has been shown to increase apoptosis in both human microglial cells and neuronal like cells of neonatal rats (16–18). Furthermore, animal studies support long-term negative effects on behavior and brain function following the administration of morphine to neonatal rats. Pups that received morphine from day 1–7 of life showed retarded motor development and as adults had impaired motor coordination, altered gait, and altered patterns of activity in an open field (19).

In preterm neonates, the use of intermittent boluses of morphine for analgesia increased the rate of the composite primary outcome of neonatal death, severe intraventricular hemorrhage (IVH), and periventricular leukomalacia (NEOPAIN trial) (20). In a separate randomized control trial, continuous infusion of morphine in preterm infants decreased the incidence of IVH but did not affect neurodevelopmental outcomes (21). Even though the preterm infant is a different population, the concern for deleterious effects of morphine infusions on the developing brain are still applicable to the near term and term population.

### Dexmedetomidine Is a Potent α_2_-Adrenergic Receptor Agonist That May Be a Better Alternative to Morphine for Newborns With HIE Treated With TH

Dexmedetomidine provides sedation, analgesia, and prevents shivering but does not suppress ventilation (22, 23). Importantly, dexmedetomidine demonstrates neuroprotective properties in several injury models including ischemia-reperfusion, anesthesia, inflammation and traumatic brain injury (24–29).

### Dexmedetomidine Is Neuroprotective in Preclinical Studies

Dexmedetomidine is a highly protein-bound drug that can readily cross the blood–brain barrier as shown in pre-clinical animal studies (30, 31). Neonatal animal models of HIE have shown that dexmedetomidine acts as potent neuroprotector via stimulation of the α-2A adrenoreceptors (32).

Exposure to dexmedetomidine following perinatal HIE reduced cortical and white matter lesion sizes and was associated with improved neurologic functional deficit (33, 34). Other possible mechanisms of dexmedetomidine neuroprotection in HIE include reducing oxidative stress and inflammation in hippocampus (32). Similarly, following controlled cortical impact, adult mice that received 3 days of dexmedetomidine therapy had decreased brain edema, inflammation, secondary blood-brain barrier damage and apoptosis (30). Following subarachnoid hemorrhage in rats, dexmedetomidine reduced brain edema and apoptosis, and improved neurological outcome scores (29).

HIE pathophysiology in the newborn shares several of these apoptotic and inflammatory pathways, therefore dexmedetomidine may have an important role for neuroprotection following HIE in neonates.

### Dexmedetomidine Is Neuroprotective in Adult Clinical Studies

In human studies, a recent meta-analysis assessed the neuroprotective effects of dexmedetomidine on ischemic brain injury in adults. Nineteen RCTs including 879 patients were analyzed and showed that compared with placebo, dexmedetomidine reduced the release of inflammatory mediators and neuroendocrine hormones and maintained intracranial homoeostasis, alleviating ischemic brain injury (35).

### Dexmedetomidine Use in a Newborn Piglet Model of HIE Undergoing Hypothermia

The PK of dexmedetomidine administered to 9 newborn piglets following HIE was reported in a de-escalation dose study (loading dose: 1 μg/kg; maintenance infusion: from 10 down to 0.6 μg/kg/h). Clearance was reduced by 32.7% at a temperature of 33.5°C and by 55.8% following hypoxia-ischemia. Importantly, high plasma levels of dexmedetomidine (>1 μg/l) were associated with cardiovascular complications (36).

### Dexmedetomidine PK in Neonates With HIE Receiving TH

Recently McAdams et al. evaluated PK and safety of dexmedetomidine in a phase I, single-center, open-label study in 7 neonates ≥ 36 weeks GA diagnosed with moderate-to-severe HIE. Infants received a continuous dexmedetomidine infusion during TH and the 6 h rewarming period. In cooled infants with HIE, clearance was either comparable or lower, distribution volume was larger, and elimination half-life was longer compared to corresponding estimates previously reported for normothermic newborns without HIE as shown in Table 1. Plasma concentrations in cooled newborns with HIE rose at a slower rate in the initial hours of infusion while similar steady-state levels were achieved. There were no adverse events (AEs) associated with dexmedetomidine treatment. The authors concluded that dexmedetomidine appeared safe for neonates with HIE at infusion doses up to 0.4 μg/kg/h. However, to overcome the initial lag in rise of plasma dexmedetomidine concentration a loading dose strategy is strongly suggested and therefore included in the present study (37).

**Table 1:** Dexmedetomidine pharmacokinetics in neonates.

	**McAdams (37)**	**Chrysostomou (38)**	**Greenberg**
Gestational Age (weeks)	39.6 ± 1.4	39.1 ± 1.6	39 (27–40)
Postnatal Age (days, weeks)	1.7 ± 0.5 d	2.23 ± 1.60 weeks	6.14 (0.57–29) weeks
Weight	3.51 ± 0.54	3.40 ± 0.60	4.02 (2.00–6.00)
Dexmedetomidine infusion rate (μg/k/h)	0.4	0.2	0.5–2.5 (max)
*C*_max_ (pg/mL)	537 ± 180	968 ± 1011^a^	304 ± 49
*T*_max_ (h)	31.0 ± 16.8	NR	End of infusion
AUC _0−∞_ (ng/mL.h)	28.4 ± 8.6	NR	NR
CL (L/h/Kg)	0.761 ± 0.155	0.907 ± 0.502	1.23 ± 0.08
MRT (h)	6.84 ± 3.20	4.26 ± 3.90	1.24 ± 0.09^*^

*Mean ± SD or median- NR, not reported; C_max_, maximum observed plasma concentration; T_max_, time of C_max_; AUC_0−∞_, area under the plasma concentration-time curve from time 0 to infinity; CL, clearance; MRT, mean residence time*;

* *p < 0.05 for a 2-tailed, 2-sample t-test between reported values for normothermic, non-HIE newborns and observed values for cooled newborns with HIE*.

### The Safety and Potential Benefits of Dexmedetomidine in Infants With HIE and Multiorgan Failure Needs Further Evaluation

In neonates, side effects of dexmedetomidine appear to be dose dependent and are mainly restricted to hemodynamic alterations including hypertension, bradycardia, and hypotension secondary to pre- and postsynaptic α_2_-receptor activation, which causes vasoconstriction, vasodilatation, and reflex bradycardia (39–41). Of importance to the present proposal is that dexmedetomidine can affect thermoregulation by affecting vasoconstriction and non-shivering thermogenesis by lipolysis, both key mechanisms present in the newborn infant. Thus, neonates with HIE undergoing TH and receiving dexmedetomidine may be vulnerable to abnormal temperature regulation (42, 43).

The short-term safety of dexmedetomidine used for sedation in 19 term neonates undergoing TH for HIE showed that it did not impact heart rate, mean arterial blood pressures, or cerebral saturations. There was no increase in extubation failures or need for vasopressors (44).

The impact of dexmedetomidine vs. morphine on cerebral blood flow and clinical outcomes was also evaluated in 205 term neonates with HIE. Patients in the dexmedetomidine cohort (*n* = 46) were extubated sooner, required lower dose of inotropes, had decreased seizure burden and lower incidence of abnormal neuroimaging when compared to the control group (*n* = 159) (45). Other benefits included shorter time to full oral feedings following TH and decreased need for NG at time of discharge (44). In a retrospective, observational cohort study including neonates with HIE undergoing TH, Cosnahan et al showed that pain scores, hemodynamics, and time to enteral feeds were similar between dexmedetomidine (*N* = 35) and morphine patients (*N* = 35). Dexmedetomidine patients received more breakthrough morphine but less cumulative morphine, and morphine patients on invasive ventilation required increased ventilatory support.

The authors concluded that dexmedetomidine seemed effective and safe for sedation and analgesia during TH since It reduced total opioid usage without increased incidence of AEs (46). In summary, data from clinical studies using dexmedetomidine in newborn infants is emerging and indicates that dexmedetomidine seems to be a safe sedative agent with possible neuroprotective effects.

### Dexmedetomidine Is Increasingly Used in Infants Undergoing TH for HIE but Rigorous Safety and PK Data Are Lacking

Even though there are limited data on PK, safety and efficacy of dexmedetomidine in infants with HIE, it has been increasingly used in many NICUs for pain and sedation during TH (38, 44, 46, 47). PK is likely different in this population compared to other infants due to both (1) altered physiology associated with critical illness and (2) TH (48). Furthermore, an immature or dysfunctional blood–brain barrier due to HIE may cause higher cerebrospinal fluid concentrations increasing sedative and analgesic effects (49).

### DICE Study Design

The Dexmedetomidine Use in Infants undergoing Cooling due to Neonatal Encephalopathy (DICE trial) will be a Phase II multicenter, unmasked, randomized, safety and PK trial. Dr. Baserga (PI) holds the FDA IND number 152829. The study is registered in Clinical Trials.Gov NCT04772222.

### Hypothesis

Our central hypothesis is that dexmedetomidine administered as sedative and analgesic therapy to infants ≥36 weeks gestational age with moderate to severe HIE undergoing TH and requiring analgesia and sedation will be safe and will be associated with improved short and long-term outcomes.

**To test this hypothesis**, we propose a Phase II multicenter, unmasked, randomized, safety and PK trial with the following Specific Aims:

**Aim 1: Examine safety measures in infants receiving dex-medetomidine compared to infants receiving morphine**. Need for sedation and pain management will be assessed using the Neonatal Pain, Agitation, and Sedation Scale (N-PASS) scores. Safety of dexmedetomidine will be evaluated during the first 4 days of life by documenting adverse events (hypotension, hypertension, bradycardia, cardiac arrhythmias, hypothermia, acute renal failure, liver failure, and seizures) outside of normal range for the study population.
**Aim 2: Determine dexmedetomidine PK** in this population of newborns with HIE during treatment with TH. Two opportunistic PK samples (at time of routine laboratories) and a PRN PK sample any time there is an adverse event will be obtained for measurement of Dexmedetomidine plasma concentrations.
**Aim 3: Determine the correlation between dexme-detomidine vs. morphine use with short-term outcomes** (clinical outcomes during initial hospitalization) **and on neurodevelopmental outcomes**. Battery of tests will include: (1) Generalized Movement Assessment (cerebral palsy assessment) performed 1 week after sedation is weaned off and at 3–4 months of life; (2) Hammersmith Infant Neurological Exam test performed at 3–4 and at 6–9 months of life; (3) Test of Infant Motor Performance performed at 3–4 months of life; and (4) Peabody Developmental Motor Skills test performed at 6–9 months of life. The Ages and Stages Questionnaires will also be completed by parents at the 6–9 months of life visit.

### Population

**Figure 1:**
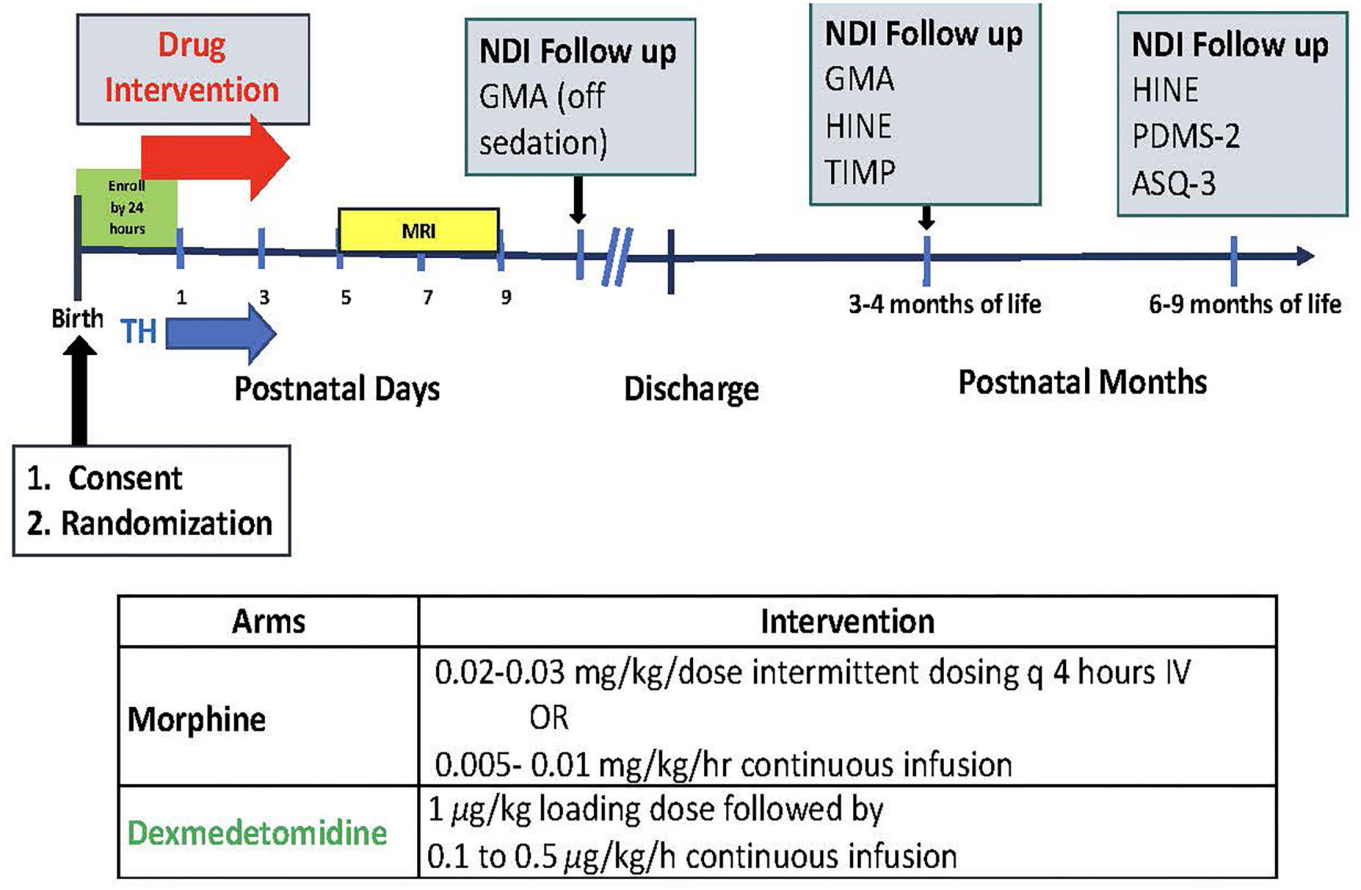
Study timeline and interventions. TH, brain MRI, neurodevelopmental impairment (NDI) follow up with GMA (Generalized Movement Assessment); HINE (Hammersmith Infant Neurological Exam); TIMP (Test of Infant Motor Performance); PDMS-2 (Peabody Developmental Motor Skills); ASQ-3 (The Ages and Stages Questionnaires).

The DICE trial will enroll 50 infants (*n* = 25/arm) ≥ 36 weeks gestational age with HIE and requiring pain treatment and/or sedation. Infants will be randomized to receive either dexmedetomidine or morphine. Figure 1 depicts the study timeline and interventions.

**Inclusion/exclusion Criteria:** Eligible neonates need to be ≥36 weeks' GA diagnosed with moderate-to-severe HIE and treated with TH (target temperature 33.5°C) for a planned duration of 72 h. To be considered for the study, infant needs to require analgesia, sedation and/or treatment to prevent shivering during TH as assessed by the N-PASS scores and a modified Bedside Shivering Assessment Scale (50, 51).

Exclusion criteria include known chromosomal anomalies; cyanotic congenital heart defects and other major congenital anomalies, as well as redirection of care being considered (not expected to survive >24 h).

Parental consent, randomization and initial treatment need to occur within 24 h of birth and as close to the initiation of TH as possible. The rationale for early intervention to treat pain and provide sedation, as well as potentially provide adjunctive neuroprotection, is based on the notion that in the early phases following HIE, dexmedetomidine could complement hypothermia through diverse mechanisms of action including anti-inflammatory and anti-apoptotic properties.

The infrastructure for this proposal consists of 5 large Level III-IV NICUs in Utah with 4,000 admissions a year. These centers have a long tradition in clinical research and participate in the Neonatal Research Network (NRN): University of Utah Hospital; Intermountain Medical Center; Primary Children's Hospital; McKay-Dee Hospital; and Utah Valley Hospital. Cumulatively, a total of 50–60 infants receive TH for HIE at these 5 participating NICUs each year. Our historical consent rate for interventional RCTs is ~60%. Including all 5 centers would allow us to complete the study in 2 years. Institutional Board Review has been obtained in 2 of the 5 centers and is in process at Intermountain Health System (3 remainder hospitals).

### Intervention

Infants will be randomized to receive either dexmedetomidine (1 μg/kg for loading dose followed by 0.1–0.5 μg/kg/h continuous infusion) or morphine (0.02–0.03 mg/kg/dose intermittent dosing q 4 h IV or as continuous infusion dose of 0.005–0.01 mg/kg/h) (37) using a REDCap centralized randomization model.

**Figure 2:**
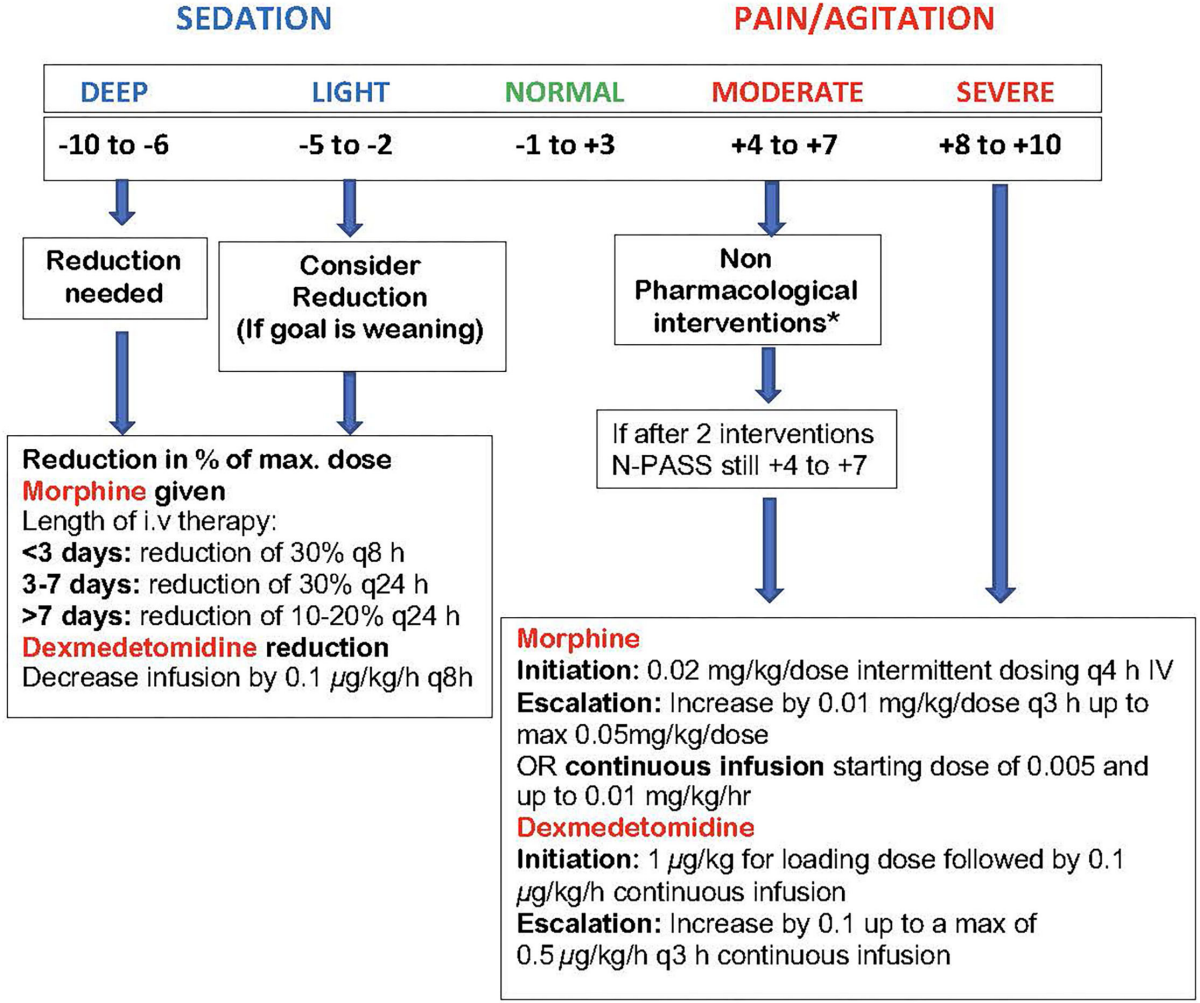
DICE trial N-PASS Assessment for Pain, Agitation and Sedation. Performed every 3 h and after 30 min post escalation or de-escalation of medication. The N-PASS tool uses *5 assessment criteria* (crying/irritability, behavior/state, facial expression, extremities/tone, and vital signs); each criterion is graded 0, −1, or −2 for sedation and 0, 1, or 2 for pain/agitation. · N-PASS score >3 (significant pain or agitation) = supplemental sedation or analgesia therapy considered. · N-PASS score >8 = initiate/escalate therapy. · N-PASS score <-2 (light sedation) = consider therapy weaning. · N-PASS score <-5 = weaning warranted. ^*^Non-Pharmacological Interventions (maybe limited during hypothermia): Nonnutritive sucking, swaddling, change of position, Kangaroo care.

Initial study drug dose will be given as soon as possible after randomization and no later than 24 h of age. Initiation and adjustment of the dexmedetomidine and morphine doses (i.e., increasing, decreasing or holding the dose) during TH will be standardized using the N-PASS scores prior to and during study drug exposure as shown in Figure 2 to determine sedation and analgesia effectiveness of both drugs (50, 52).

### Outcomes

The primary outcome of this study will be safety. Secondary outcomes will include PK and short and long-term clinical outcomes including clinical course during the initial hospitalization (incidence of shivering, respiratory support, time to full oral feedings, seizure burden, etc.) as well as neurodevelopmental follow up (up to 9 months of age).

### Safety

We will determine safety in infants receiving dexmedetomidine compared with infants receiving morphine during the first 4 days of life by documenting AEs such as hypotension, hypertension, acute renal failure, liver failure, poor cardiac function, cardiac arrhythmias, respiratory failure, hypothermia and seizures outside of normal range for the study population (9, 53). This population includes critically ill infants that have morbidities associated with HIE that can overlap or potentiate some of the side effects of TH, morphine and dexmedetomidine. We will therefore compare morbidities between the 2 interventional groups (morphine vs. Dexmedetomidine) and with historical cohorts of infants that underwent cooling in large RCTs (4–6, 9, 53). We will consider AEs those that are outside of normal range expected for the study population.

Severe adverse events (SAEs) will be defined as any event that results in death, a life-threatening event, a persistent or significant disability that prolongs inpatient hospitalization. SAEs will be reported until 30 days of life or hospital discharge, whichever occurs first.

Criteria for withholding or stopping the study drug will be the occurrence of an AE requiring intervention: severe bradycardia (heart rate <60 sustained for >30 min); serious cardiac arrythmias (atrial fibrillation); severe hypotension (<30 mm Hg MBP) or hypertension (sustained SBP >95th centile) requiring treatment; and severe hypothermia (esophageal temperature <31°C). An independent Data Safety Monitoring Committee (DSMC) composed by members qualified by training and experience to monitor the progress of the investigation will monitor study progress, safety and efficacy. An interim safety analysis will be performed after 10 infants are enrolled. The trial will be temporarily suspended for the initial safety review and as deemed necessary by the DSMC for any subsequent safety concerns.

**Pharmacokinetics**: We will determine dexmedetomidine PK in this population of neonates with HIE during treatment with TH and during rewarming periods (Day 1–4). To avoid excessive phlebotomy losses, a sparse sampling strategy will be employed. Two opportunistic blood samples (0.1 mL) will be obtained for each participant at the time of routine laboratories. Additional PK samples will be collected any time there is an AE that is at least possibly related to dexmedetomidine.

The rich sampling strategy employed in adult PK studies is rarely feasible in infant PK studies due to barriers to informed consent and limited blood volume. A sparse sampling strategy when combined with modeling approaches such as population PK and physiologically-based PK have successfully characterized the PK in infants and children and this approach is recommended by regulatory agencies for pediatric studies (54–61).

Plasma will be frozen at −80°C until shipped to the Bioanalytical Core at Children's Hospital of Philadelphia for analysis. Dexmedetomidine concentrations in plasma will be quantified using an existing, validated HPLC-MS/MS assay. Accuracy and precision are within the FDA bioanalytical assay validation criteria (±15%). This method was employed in a dexmedetomidine PK study in neonates after cardiac surgery (62). Dexmedetomidine plasma PK data will be analyzed with a non-linear mixed effects modeling approach using NONMEM (version 7.4, Icon Solutions, Ellicott City, MD, USA).

### Clinical and Neurodevelopmental Outcomes

To determine the extent to which dexmedetomidine is associated with improved short-term clinical outcomes during initial hospitalization the following data will be collected:

1. **Cardiovascular**: Severe Hypotension (Sustained Mean Blood Pressure <30 mm Hg MAP) or Hypertension (Sustained Systolic Blood Pressure >95th Centile), Bradycardia/Tachycardia, Need for Inotropes, Need for Inhaled Nitric Oxide, and Echocardiogram Results.
2. **Respiratory Support**: Need for Nasal Cannula, CPAP, Invasive Mechanical Ventilation, Time to Extubation, Extubation Failure. Ventilatory Settings and Oxygen Requirements Will be Recorded.
3. **Shivering**: Will be Assessed Every 3 h With Other Vital Signs by the Bedside Nurse Using an Adapted Version of the Bedside Shivering Assessment Scale (51).
4. **Other**: Time to Full PO Feedings; Need for Gastric Tube/NG Feedings at Discharge; Seizure Burden (Total Duration of Seizures in first 24 h of Life); Neurologic Exam (at 7 Days of Life or at Discharge); Magnetic Resonance Imaging, Head Ultrasound; EEG Results.

### Neurodevelopmental Follow up

All HIE infants that receive TH automatically qualify for follow up by the University of Utah Neonatal Follow Up Program and Pediatric Neurology. The following well established tests will be used: (1) Generalized Movement Assessment (GMA) (cerebral palsy assessment) performed 1 week after sedation is weaned off and at 3–4 months of life; (2) Hammersmith Infant Neurological Exam (HINE) test performed at 3–4 and at 6–9 months of life; (3) Test of Infant Motor Performance (TIMP) performed at 3–4 months of life; and (4) Peabody Developmental Motor Skills (PDMS-2) test performed at 6–9 months of life. These validated tests are core components of the neurodevelopmental assessment for early diagnosis of cerebral palsy (63–65). In the present proposal, these tools will be used as markers of neurodevelopmental safety following the use of dexmedetomidine. The Ages and Stages Questionnaires (ASQ-3) will be completed by parents at the 6–9 month visit to screen infants in areas of communication, gross and fine motor, and personal-social (66). This data will be used to power a Phase III dexmedetomidine neuroprotective efficacy trial.

## Sample Size and Statistical Analyses

For the **sample size calculation**, 50 subjects (25 in each arm) would yield 80% power to compare dexmedetomidine relative to morphine for key safety measures using tolerance limits of 0.5 SDs for quantitative measures of organ function. The safety markers and clinical meaningful changes that we have identified to calculate the current power statement include the incidence of hypotension, bradycardia, persistent pulmonary hypertension (PPHN), renal adverse events, and hepatic dysfunction in both groups. The incidence of hypotension that required used of inotropes (40%), significant bradycardia (5%), PPHN (15%), renal adverse events (41%) and liver dysfunction (32%) has been reported in previous cooling cohorts and will be used as baseline (53).

This sample size is also considered adequate to evaluate clinical (hospitalization) and neurodevelopmental impairment as secondary outcomes. Lastly, the sample size of 50 is adequate to evaluate feasibility for a potential phase III trial that will be powered for long term outcomes.

For the analysis of safety effect, we will use an intention-to treat strategy. To compare AEs and SAEs counts between treatment groups a Poisson regression model with robust SEs adjusting for the severity of encephalopathy (moderate vs. severe) will be used. To calculate the effect of dexmedetomidine vs. morphine on developmental outcome measures, we will use 95% confidence intervals, and corresponding *P*-values (significance, *P* < 0.05) using a generalized linear model with robust SEs, and appropriately adjusting for patient covariates using an all subsets regression model with corrected Akaike information criterion (AICc). For summary statistics, we will compare categorical variables (GA, birthweight, gender) between randomized treatment groups by using a χ_2_ test, or Fisher's exact test when the expected count of any category is ≤5. To compare baseline continuous variables between treatment groups, a two-sided *t*-test will be used.

### Limitations

We expect that a proportion of the DICE subjects will be transferred from referring institutions, challenging the time-sensitive consent process. For successful recruitment (window to obtain informed parental consent is 24 h) each site will follow a dedicated screening process to alert the research team of new eligible admissions. Our sites have significant experience in approaching families of potential subjects in a timely fashion as most RCTs require approaching parents within hours of admission to the NICU (67, 68). For instance, in the “Darbepoetin administration to neonates undergoing cooling for encephalopathy: a safety and pharmacokinetic trial (DANCE)” our randomization time was a mean of 8 h of life in 30 infants demonstrating feasibility of such studies in this sick population (68).

Lost to follow up (about 10% in our 5 centers) is another potential difficulty. To address this issue, we have a dedicated follow up research coordinator that maintains periodic contact with the participating families. Another advantage is that HIE/cooled infants automatically qualify for follow up in the University Developmental Assessment Clinics as well as with Pediatric Neurology at the same age as the NDI follow up is planned for the DICE study (3–4 months and 6–9 months of life).

Although not ideal, the design of the DICE trial will be unblinded due to the fact that masking a loading dose followed by continuous Dexmedetomidine infusion vs. Morphine to be given as intermittent dosing q 4 h IV or (less likely) as a continuous infusion would be challenging. Many centers use morphine as a PRN bolus drug so allocating all babies in the morphine arm to receive a continuous infusion would not be generalizable. For infusion therapies blinding can translate into a cumbersome and costly setup (69). Lastly, this is a Phase 2 safety and PK trial where blinding perhaps is not as crucial as in a phase III efficacy study in which masking key personnel in charge of assessing outcomes (bedside nurse obtaining pain scores, neurodevelopmental assessors, etc.) would be key.

## Conclusions/Discussion

HIE remains an important cause of death and adverse neurological outcome. Because of the vulnerability of the brain following HIE, avoiding the use of potentially harmful drugs for analgesia and sedation such as morphine and replacing it with a potentially safer drug such as dexmedetomidine may further reduce brain injury. However, inappropriate dosing places these infants at risk for therapeutic failure and toxicity (49). Further research to determine safe and effective therapies for the treatment of pain and stress during hypothermia is essential to guide the development of clinical treatment protocols. The DICE trial will hopefully advance the knowledge about the safety and PK of dexmedetomidine in critically ill neonates with HIE undergoing TH. The PK and outcome data obtained from this trial will be used to inform larger Phase III trials with long-term neurodevelopment assessments at 2 years of age and school age as the primary outcome.

## Data Availability Statement

The original contributions presented in the study are included in the article/Supplementary Material, further inquiries can be directed to the corresponding author/s.

## Author Contributions

KW, MS, BO, and TD contributed to the study design and manuscript preparation. AB and TB contributed to the statistical study design and manuscript preparation. MB contributed to the study conception and design, and manuscript preparation. All authors read and approved the final manuscript.

## Conflict of Interest

The authors declare that the research was conducted in the absence of any commercial or financial relationships that could be construed as a potential conflict of interest.

## Publisher's Note

All claims expressed in this article are solely those of the authors and do not necessarily represent those of their affiliated organizations, or those of the publisher, the editors and the reviewers. Any product that may be evaluated in this article, or claim that may be made by its manufacturer, is not guaranteed or endorsed by the publisher.
